# Functional relevance of naturally occurring mutations in adhesion G protein-coupled receptor ADGRD1 (GPR133)

**DOI:** 10.1186/s12864-016-2937-2

**Published:** 2016-08-11

**Authors:** Liane Fischer, Caroline Wilde, Torsten Schöneberg, Ines Liebscher

**Affiliations:** From the Section of Molecular Biochemistry, Institute of Biochemistry, Medical Faculty, University of Leipzig, Johannisallee 30, 04103 Leipzig, Germany

**Keywords:** GPR133, ADGRD1, Adhesion GPCR, Mutations, SNP, Database

## Abstract

**Background:**

A large number of human inherited and acquired diseases and phenotypes are caused by mutations in G protein-coupled receptors (GPCR). Genome-wide association studies (GWAS) have shown that variations in the *ADGRD1* (*GPR133*) locus are linked with differences in metabolism, human height and heart frequency. ADGRD1 is a G_s_ protein-coupled receptor belonging to the class of adhesion GPCRs.

**Results:**

Analysis of more than 1000 sequenced human genomes revealed approximately 9000 single nucleotide polymorphisms (SNPs) in the human *ADGRD1* as listed in public data bases. Approximately 2.4 % of these SNPs are located in exons resulting in 129 non-synonymous SNPs (nsSNPs) at 119 positions of *ADGRD1*. However, the functional relevance of those variants is unknown. In-depth characterization of these amino acid changes revealed several nsSNPs (A448D, Q600stop, C632fs [frame shift], A761E, N795K) causing full or partial loss of receptor function, while one nsSNP (F383S) significantly increased basal activity of ADGRD1.

**Conclusion:**

Our results show that a broad spectrum of functionally relevant *ADGRD1* variants is present in the human population which may cause clinically relevant phenotypes, while being compatible with life when heterozygous.

**Electronic supplementary material:**

The online version of this article (doi:10.1186/s12864-016-2937-2) contains supplementary material, which is available to authorized users.

## Background

More than three dozen human inherited and acquired diseases are caused by mutations in G protein-coupled receptors (GPCRs) [1–4]. At the molecular level, mutational alteration in GPCRs can have two major consequences – the gain and loss of receptor function. Most mutations causative for human diseases have been found in rhodopsin-like GPCRs, which comprise the largest family of GPCRs, but only a few mutations in the second largest family; the adhesion GPCRs (aGPCRs). Examples are ADGRG1 (GPR56) and ADGRV1 (VLGR1), where gene mutations cause brain malformation (bilateral frontoparietal polymicrogyria) [5] and a form of Usher syndrome [6], respectively.

ADGRD1, a member of the aGPCR family, was among the first aGPCRs identified in genome-wide association studies (GWAS) where SNPs in intronic regions were associated with human height in Sorbs and Korean populations [7, 8]. A GWAS with over 1000 subjects revealed an association of *ADGRD1* with the postprandial levels of very low density lipoproteins (VLDL) [9]. Marroni et al. found two SNPs associated with RR duration leading to an increase of one beat per second in heart rate [10]. Recently, the methylation status of the *ADGRD1* locus was associated with body weight in humans [11] which is in agreement with an earlier study connecting SNPs in this gene and body weight in mice [12].

**Fig. 1:**
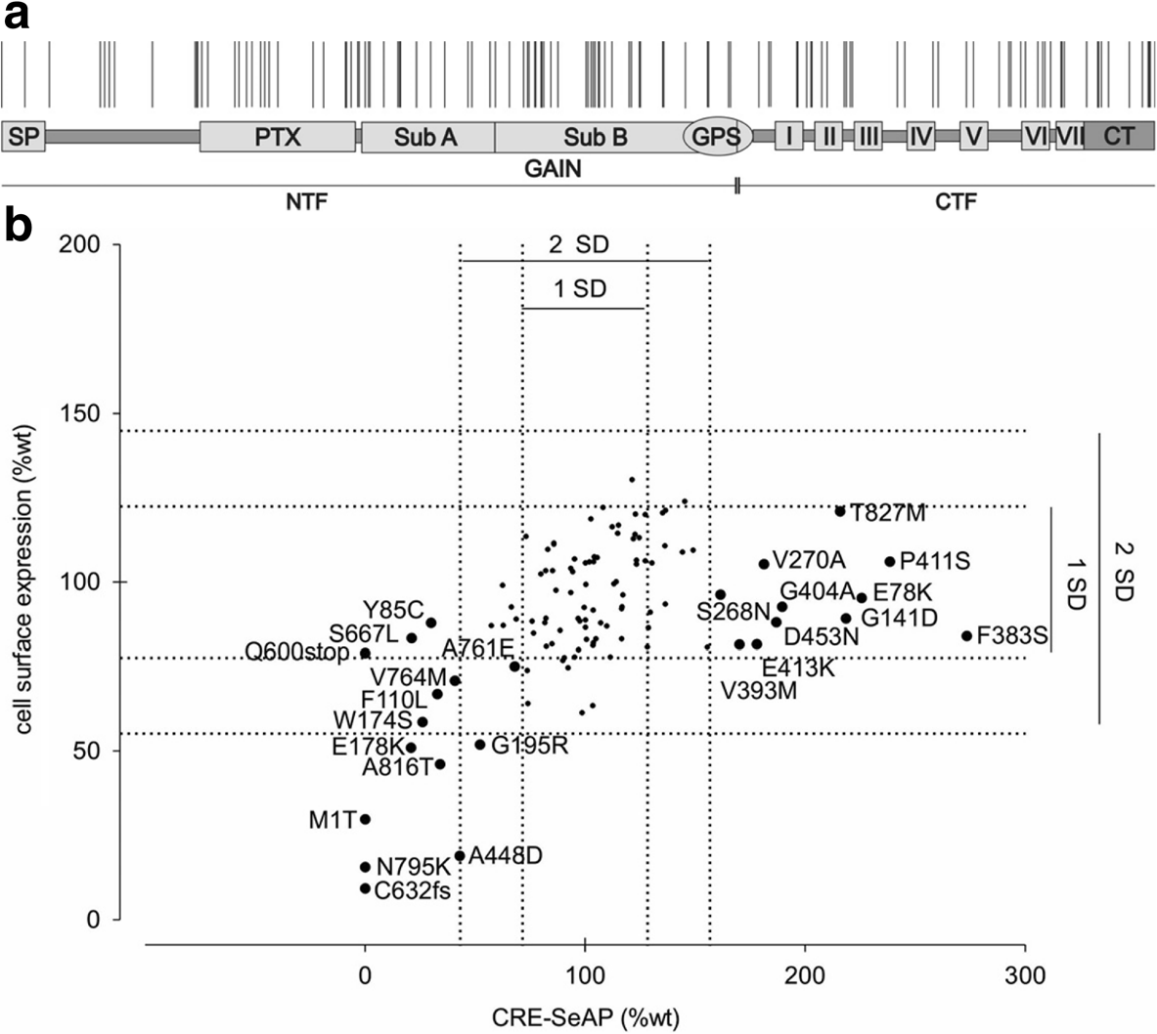
nsSNPs in *ADGRD1* influence cell surface expression and cAMP responsive element activity. **a** Schematic amino acid structure of ADGRD1 which consists of 874 amino acids. The signal peptide (SP) is followed by a pentraxin domain (PTX), the GPCR autoproteolysis inducing (GAIN) domain (including the two subdomains) harboring a GPCR proteolysis site (GPS), the 7TM region (I-VII) with 3 intra and 3 extracellular loops and a C-terminal tail (CT). The receptor is divided into two fragments: an N-terminal fragment (NTF) and a C-terminal fragment (CTF) after autoproteolytic cleavage at the GPS. Each vertical line indicates a position influenced by amino acid changing single nucleotide variants (data collection: 21-04-2015). **b** Each dot represents the mean of one inspected nsSNPs in ADGRD1 (for details see Additional file 1). The dotted lines indicate the one- and twofold standard deviation (SD) from wt in CRE-SeAP activity. Data are given as % of wt activity and cell surface expression as mean ± SD. Empty vector (EV) served as negative control (basal activity: EV: 568,311 ± 59,100 counts; wt: 786,125 ± 85,787 counts; *n* = 4; basal expression: EV: 0.01 ± 0.02 OD_492 nm_; wt: 0.70 ± 0.11 OD_492 nm_; *n* = 12)

Our data base analysis of more than 1000 sequenced human genomes including the 1000 Genomes [13], exome sequencing projects (NHLBI-ESP [14], ClinSeq Project [15]) and the human haplotype map [16], revealed approximately 9000 single nucleotide polymorphisms (SNPs) in the human *ADGRD1* gene. Out of those 2.4 % SNPs are located in exons leading to 129 amino acid changes at 119 positions (Fig. 1a). Compared to other GPCR genes [17] the number of SNPs in *ADGRD1* is not significantly different. However, a functional relevance of most SNPs can only be evaluated by experimental testing. In general, their large size, the lack of agonists and knowledge about their signal transduction, until recently, rendered the functional characterization of aGPCRs very difficult.

ADGRD1, like most members of the aGPCR family, displays a characteristically complex and long N terminus. The ectodomain contains a pentraxin-like (PTX) domain (as a part of the Laminin G3 domain (LamG3)) as well as a GPCR autoproteolysis inducing (GAIN) domain. The structure of the GAIN domain, which also harbors the GPCR proteolysis site (GPS), has been resolved [18], showing that it is divided into two subdomains: the up to six α-helices containing subdomain A and the 13 β-strands containing subdomain B. A proposed autoproteolytic event at the GPS [19] cleaves the full length receptor protein in an extracellular N-terminal fragment (NTF) and an intracellular C-terminal fragment (CTF), which contains the seven transmembrane region [20, 21].

We have recently shown that ADGRD1 couples to the G_s_ protein/adenylyl cyclase signaling pathway [22] and is activated by a tethered agonist [23]. This now allows for characterization of natural *ADGRD1* variants found in the human population. We identified several inactivating and constitutively activating mutations in the human *ADGRD1* gene. This will provide the molecular basis of understanding phenotypes related to *ADGRD1* variants and will initiate in-depth clinical characterization of individuals carrying such receptor variants.

## Results and discussion

### Allelic variation is comparable to rhodopsin-like GPCR and variations can occur in all parts of ADGRD1

According to NCBI database of Single Nucleotide Polymorphisms (dbSNP) nearly 9000 single nucleotide polymorphisms were identified in the *ADGRD1* gene (*ADGRD1*, Gene ID: 283383, chromosomal position [GRCh38 Primary Assembly, human Chr 12, NT_029419.13] 130953907..131141465) [24]. Approximately 96 % of SNPs are intronic, while the remaining 4 % cluster in untranslated regions (UTR) and exons. The SNPs within exons led to 88 synonymous changes and 129 non-synonymous exchanges including 3 frameshifts and 1 stop mutation (Table 1). Additional file 1: Table S1 summarizes characteristics and origin of 109 nsSNPs that were analyzed in this study. *ADGRD1* displays a SNP frequency of ~24.8/100 codons, which is comparable to those of rhodopsin-like GPCRs (Table 1) [17]. Notably, there is no ‘hotspot’ of nsSNPs within the coding region (Fig. 1a). One should consider that sequencing errors might be responsible for some of the listed nsSNPs and that all functionally relevant mutations occurred only heterozygously at low frequency in the populations investigated. However, reevaluation of sequencing data of some inactivation mutations revealed considerable coverage of the detected nsSNPs in those individuals (personal communication Dr. Biesecker), thereby verifying some of the most striking variants.

**Table 1:** Comparison of exon SNPs of human *ADRGD1* with Rhodopsin-like GPCRs

	Human *ADGRD1* ^a^		Rhodopsin-like GPCRs (Stäubert et al., 2014 [17])
	Absolute n	n/100 codons	n/100 codons
Synonymous	88	10	10
Non-synonymous	129	15	15
Frameshift	3	0.3	0.3
Stop mutation	1	0.1	0.5

^a^ Data collection: 21.04.2015

### nsSNPs in ADGRD1 change receptor activity on a broad scale

Receptor function can be evaluated at the levels of signaling activity and of membrane expression, latter as a measure of proper folding and translocation. For example, most of the loss-of-function mutations in the vasopressin type 2 receptor, which lead to nephrogenic diabetes insipidus, display significant reduction of cell surface expression [25]. Gain-of-function GPCR mutants exhibit mostly lower cell surface levels as a result of reduced phosphorylation, structural instability and enhanced internalization and degradation but show simultaneously significant increases in basal signaling activity [4, 26–28].

ADGRD1 is a G_s_ protein-coupled receptor with high basal activity and high expression levels at the cell surface [22, 23, 29]. To efficiently evaluate the functional relevance of the natural occurring nsSNPs we initially took advantage of the high basal activity of ADGRD1 as a sensitive measure for its G_s_-protein coupling ability. Thus, we examined 109 nsSNPs in an assay that uses cAMP response element (CRE) to trigger the secretion of an alkaline phosphatase (CRE-SeAP assay) to assess signaling activity, while cell surface and whole cell expression was monitored in parallel through ELISAs (Additional file 2). Supported by experiences with other GPCRs [1–4] we considered the combination of these assays to be sufficient to identify most mutants with impaired or significantly increased basal activity and/or altered cell surface trafficking. Based on the function (basal CRE levels) and expression levels of the human wt ADGRD1 a twofold standard deviation (SD) was set as arbitrary threshold to define “normal” function. This is a reasonable range as shown in studies evaluating the functional distribution of GPCR orthologs [30, 31].

Except for two nsSNPs (M1T, N795K), all examined nsSNPs displayed very similar total cell expression levels compared to wt ADGRD1 (Additional file 2). To identify those nsSNPs, which cause alteration of basal CRE-level and/or of cell surface expression, all variants were plotted in respect to these parameters (Fig. 1b). Almost 60 % of the analyzed receptor mutants exhibited wt CRE-SeAP levels, 12.5 % showed more than and 11.6 % less than 2-fold SD different CRE-response from wt (Fig. 1b; Additional file 2). The remaining variants were within the 1-fold SD. With 80 % of the variants displaying wt cell surface expression levels this parameter was less effected (Fig. 1b; Additional file 2). Activity-altering nsSNPs were found evenly distributed throughout the receptor (Table 2).

**Table 2:** Domain distribution of functional relevant nsSNPs

	AA	Inspected nsSNPs	±2SD
	n	n	n (% inspected nsSNPs)
Whole receptor	874	107	23 (21.5)
N terminus	570	76	17 (22.4)
PTX	111	15	3 (20.0)
GAIN	295	43	9 (20.9)
7TM	243	21	4 (19.0)
C terminus	61	10	2 (20.0)

Exclusion of stop and frameshift mutants

### nsSNPs within ADGRD1 change basal activity levels in a domain-specific manner

**Fig. 2:**
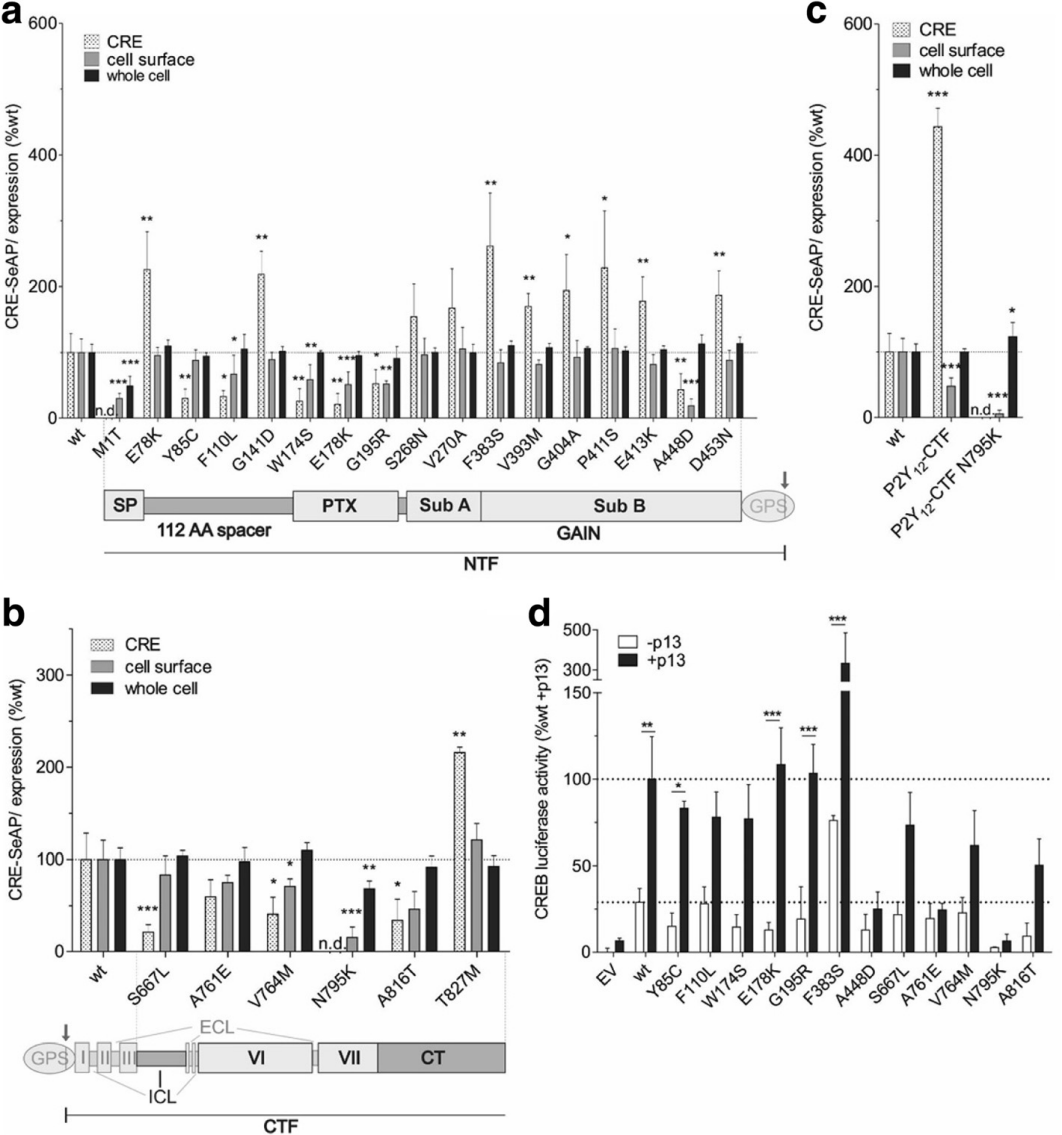
nsSNPs in *ADGRD1* influence signaling activity in compartment-depending manner. **a** N-terminal nsSNPs of *ADGRD1* have different influences on signaling activity and expression. The positions of nsSNPs within the N-terminal fragment (NTF) are indexed through the pictogram under the graph. **b** nsSNPs in 7TM core and C terminus influence cAMP response element (CRE)-signaling activity. The schema under the graph indicates the position of signaling activity relevant nsSNPs: S667L in intracellular loop (ICL) 2, V764M in TM6 (VI), N795K in TM7 (VII) and A816T and T827M in C terminus. **c** Constitutive activity of P2Y_12_-CTF-mutant is lost after insertion of N795K. (A-C) Graphs show the percentage of wildtype (wt) after normalization to mock control of functional relevant nsSNPs in CRE-SeAP activity (EV: 568,311 ± 59,100 counts; wt: 786,125 ± 85,787 counts; *n* = 4), cell surface expression (EV: 0.02 ± 0.03 OD_492 nm_; wt: 0.70 ± 0.11 OD_492 nm_; *n* = 9) and whole cell expression (EV: 0.08 ± 0.07 OD_492nm_; wt: 0.97 ± 0.16 OD_492nm_; *n* = 7). To compare differences of receptor mutants to wt basal activity or expression levels an unpaired two-tailed t-test was performed with * *p* ≤ 0.05, ** *p* ≤ 0.01, *** *p* ≤ 0.001. **d** Activation analysis of nsSNPs upon stimulation with the tethered agonist-derived peptide p13. After normalization to mock control (EV: 872 ± 200 counts; *n* = 7) data are shown as percentage of wildtype stimulated with p13 (wt + p13: 5.3 ± 1.3 x-fold over EV; *n* = 7). To analyze significance of receptor mutant activation after p13 stimulation a two-way ANOVA with Bonferroni as post-test was used: * *p* ≤ 0.05, ** *p* ≤ 0.01, *** *p* ≤ 0.001. **a**-**d** Data are given as means ± SD

The majority of nsSNPs in the N terminus that alter receptor activity levels were located either at the very N terminus or within the GAIN domain (Fig. 2a). The very N terminus includes the signal peptide, a PTX-like domain (as a part of the LamG3 domain) and a 112 amino acid-long spacer region. In this receptor region, nsSNPs mainly affected the receptor expression level with a subsequent decrease in CRE activity levels. M1T (rs144264048, T > C) was found heterozygously in a European American population (Additional file 1). Interestingly, this missense mutation was expressed in whole cell and can be detected at low levels at the cell membrane. However, relevant basal CRE activity was not observed (Fig. 2a). These findings are in accordance with a report by Kozak, who found that a one-base change of AUG can function as initiator codon [32]. The efficiency for initiation of ACG, however, is under 3 % compared to AUG [32]. There have been reports of an existing AUG-ACG mutation in the human α globulin gene, which reduces gene expression resulting in clinical thalassemia [33].

Two mutations, E78K [rs267603378, G > A] and G141D [rs142759046, G > A], in the spacer region, led to significant increase in activity, which cannot be explained by an associated elevated membrane expression (Fig. 2a). E78K was identified by Y. Samuels (NHGRI/NIH; http://www.ncbi.nlm.nih.gov/clinvar/RCV000062447/) as one out of three nsSNPs within *ADGRD1* that are found in malignant melanoma tissue [34]. G141D was found in two heterozygous genotypes out of 2201 in African Americans by NHLBI ESP and by 1000 Genomes (Additional file 1).

There were two mutations, Y85C [rs199848650, A > G] and F110L [rs148928637, C > G], in the spacer region that led to significant decrease in activity (Fig. 2a). Y85C appeared in the ClinSeq study that analyzed 1306 chromosomes from 662 people of a European population. Of note, ClinSeq includes healthy and disease-affected participants and had an initial focus on cardiovascular disease [15]. Yet, the individual carrying this SNP showed no obvious clinical phenotype (personal communication Dr. Biesecker).

W174S (rs141606054, G > C), E178K (rs148148477, G > A) and G195R (rs267603379, G > A) are located within two alpha helical structures of the PTX-like domain, as predicted via hydrophobicity plot according to Kyte- Doolittle with Protean 7.1.0 (DNAStar, Lasergene). The latter two SNPs have also been identified by Samuels’ laboratory (NHGRI/NIH) as being found in samples from malignant melanoma. Amino acid changes at these positions significantly decrease cell surface expression, which results in an even more pronounced reduction of CRE levels (Fig. 2a). W174 and G195 are 100 % conserved in 74 vertebrate *ADGRD1* orthologs whereas the position E178 is substituted by aspartate in 12 % of orthologous sequences but not by lysine (Additional file 2).

The GAIN domain is considered an important structural and functional element of aGPCRs [18]. Nine out of 43 inspected nsSNPs within the GAIN domain change ADGRD1-mediated CRE levels, the majority of them leading to constitutive activation of the receptor (Fig. 2a). This is most pronounced in case of F383S (rs200232576, T > C), which increased receptor activity by 262 %. Considering that *ADGRD1* has been associated with changes in heart frequency [10] it is striking to see that the most prominent activity increasing SNP origins from a cohort that includes participants with potential heart maladies. Nonetheless, F383S is found in a single person of the ClinSeq project [15] that does not show signs of coronary artery disease (personal communication Dr. Biesecker). The position F383 is conserved and only substituted by tyrosine in orthologs (Additional file 2). Within the GAIN domain only A448D (rs200173874, C > A) reduced receptor activity most likely because of reduced plasma membrane expression. This functional relevance of A448 is in agreement with its high evolutionary conservation (Additional file 2). Interestingly, out of the six investigated nsSNPs within the highly conserved GPS region, there was none that significantly changed receptor activity.

Several nsSNPs within the highly conserved 7TM core (S667L (rs377401276, C > T) in ICL2, A761E (rs369201469 C > A) and V764M (rs149434203, G > A) in TM6 and N795K (rs369853823, C > A) in TM7) led to a reduction in receptor-function (Fig. 2b). N795K completely abolished the CRE receptor signal. Even introduced into the constitutive active P2Y_12_-CTF chimera [23], N795K completely abolished the function of this construct (Fig. 2c). N795K was found to be heterozygous in European Americans (NHLBI ESP) with a minor allele frequency of 0.000077 (Additional file 1). The position is highly conserved in vertebrates (Additional file 2).

The importance of cleavage for aGPCR function is still a matter of debate, yet GPR133-cleavage deficient mutants have been shown to display similar cell surface and cAMP levels compared to the wt receptor [22]. In line with this, a selection of activating, inactivating and control SNPs with wt function throughout the GPR133 structure did not display any changes in occurrence of cleavage (Additional file 2).

Our analysis includes data from three studies with a pathophysiological focus: (1) coronary artery disease (ClinSeq study; [15]), (2) malignant melanoma [34] and (3) heart, lung and blood disorders (the NHLBI-ESP study; [14]). So far, for none of the functionally altered variants a direct attribution to an affected individual could be found. However, two nsSNPs, with significantly reduced, and one nsSNP, with increased signaling abilities, were found in samples with malignant melanoma.

Since all variants were found in adults our data indicate that inactivation or constitutive activation of only one *ADGRD1* allele is compatible with life and does not result in a clinically relevant phenotype in humans. However, association of nsSNPs in *ADGRD1* with common human diseases cannot be excluded. Of note, the nsSNP A446V, which was previously identified by a study searching for genes influencing human height [8] displayed wt function in our assays.

### Application of the Stachel-derived peptide agonist p13 can restore wt activity levels of selected nsSNPs

To evaluate the capability of these receptor variants to be activated through the tethered peptide agonist of ADGRD1, we stimulated selected receptor mutants with 500 μM of the synthetic peptide agonist p13 [23] (Fig. 2d). As a functional read-out a luciferase based reporter gene assay under the control of CREB was used. Half of the analyzed nsSNPs showed wt-equivalent activation levels upon peptide application despite reduced basal activity levels, indicating that these mutants are capable of exerting wt function. Some nsSNPs with significantly reduced activation levels through p13 (A448D, V764M, N795K and A816T) were expressed at lower levels in the cell membrane, accounting for the observed lack in stimulation (Fig. 2a and d). However, there are also nsSNPs (F110L, W174S, E178K, G195R) which showed significantly reduced cell surface expressions but wt typical peptide responses (Fig. 2a and d). It seems that peptide stimulation can compensate lack of membrane expression as long as the mutation is within the very N-terminal region of the receptor. Of note, all nsSNPs that do not reach wt activation levels upon p13 are located either in the GAIN domain or the 7TM core. It is conceivable from this data that there are natural variations within the binding pocket of the tethered agonist, which is yet to be defined. Specifically, position A761 in TM6 seems to be an interesting candidate since the reduction in cell surface expression is not low enough to explain the deficiency in activation through p13 (Fig. 2b and d). N795K, as part of TM7 most likely disrupts proper protein folding as plasma membrane expression is severely disrupted. Therefore, neither basal nor peptide-induced activity was detected (Fig. 2c and d).

The constitutively active F383S-mutant, as a part of the second β-strand in subdomain B of the GAIN domain [18], exhibited an even more pronounced p13- induced activation (Fig. 2d). Here additive effects are likely the cause.

### Characterization of ORF-disrupting ADGRD1 variants

**Fig. 3:**
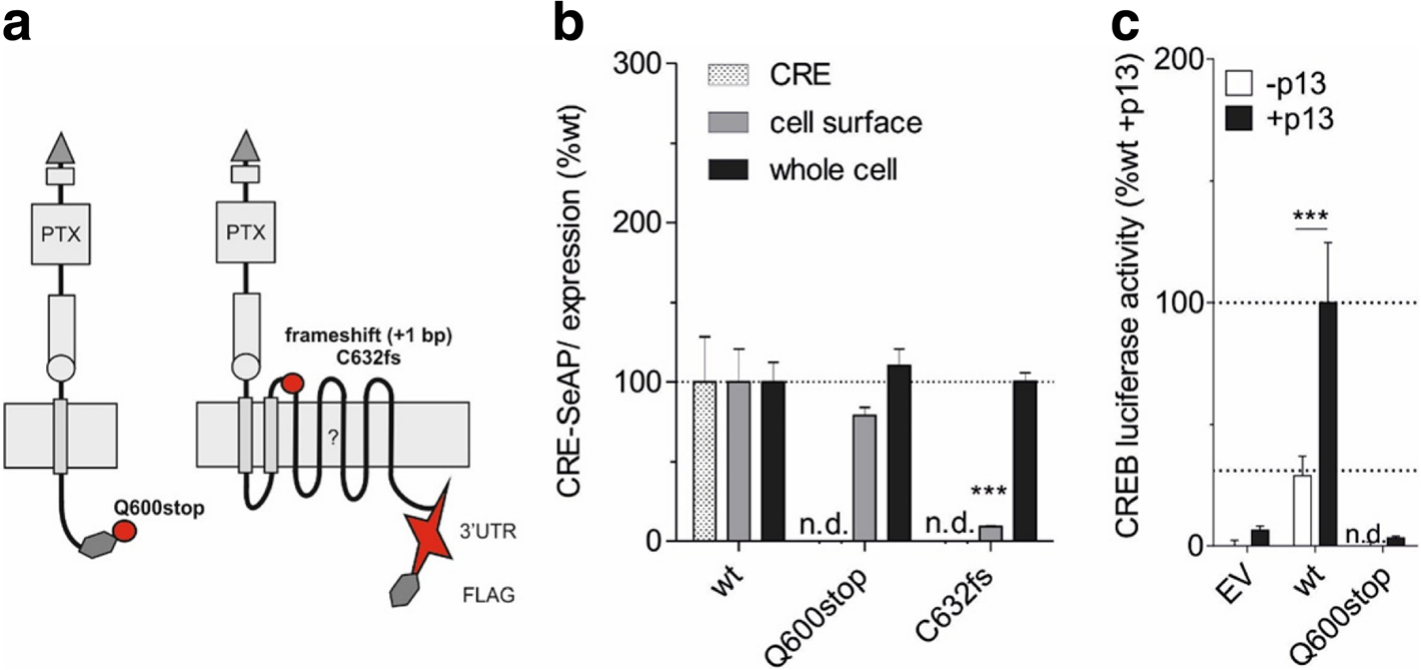
nsSNPs leading to length variations do not elicit intracellular signal transduction. **a** Schematic structure of the cloned constructs for the length variation Q600stop and the frameshift C632fs are shown in the middle. The signal peptide (dark grey triangle) is followed by an HA-tag (light grey box) and the functional domains of the NTF: a PTX (grey box labeled ‘PTX’) and GAIN domain (rectangle including GPS as circle). Transmembrane units are indicated as grey boxes downstream the GPS circle. The C terminus features a hexagonal FLAG-tag. The star indicates the newly cloned 3′UTR region. **b** Two days after transfection CRE-SeAP assay, cell surface and whole cell ELISA were performed. Graphs show the percentage of wildtype (wt) after normalization to mock control of functional relevant nsSNPs in CRE-SeAP activity (EV: 568,311 ± 59,100 counts; wt: 786,125 ± 85,787 counts; *n* = 4), cell surface expression (EV: 0.02 ± 0.03 OD_492 nm_; wt: 0.70 ± 0.11 OD_492nm_; *n* = 9) and whole cell expression (EV: 0.08 ± 0.07 OD_492 nm_; wt: 0.97 ± 0.16 OD_492 nm_; *n* = 7). **c** Stimulation with p13 could not activate Q600stop. Data are shown as x-fold over mock control (EV: CREB-Luciferase: 1320.6 ± 628.1 counts; *n* = 4). **a**-**c** Data are given as means ± SD. To compare differences of receptor mutants to wildtype basal activity or expression levels an unpaired two-tailed t-test was performed, for receptor activation after p13 stimulation two-way ANOVA with Bonferroni as post-test was used, ** *p* ≤ 0.01 *** *p* ≤ 0.001; n.d., not detectable

The natural variant Q600stop (rs200986302, C > T) leads to a premature truncation of ADGRD1 in the first intracellular loop (Fig. 3a). Nonetheless, the protein was expressed at cell surface ELISA (Fig. 3b). As expected, truncation of the last six transmembranes resulted in a complete lack of basal and peptide-induced signal transduction (Fig. 3c). This unusual SNP was also found heterozygously in a single person in the ClinSeq study. Again no clear sign of coronary artery disease was evident (personal communication Dr. Biesecker).

There is a natural variation of ADGRD1 that causes an extension of the reading frame (Fig. 3a). In C632fs [rs34176886, />C]), an insertion of one base pair in extracellular loop one leads to a frameshift which results in a 78-amino acid longer receptor. Even though it seems that this protein is produced in the cell, it lacks expression in the plasma membrane. Therefore, no second messenger activity was detected (Fig. 3b). To verify the frameshift, the Flag epitope was set into frame of this mutant right upstream of the newly gained stop codon. A pronounced signal in the sandwich ELISA indicated the translation of this frameshift-caused artificial protein (Fig. 3b).

In sum, there are at least two naturally occurring nsSNPs that lead to non-functional ADGRD1 due to gross alteration of the receptor protein chain. In contrast to classical rhodopsin-like GPCR, where truncation after the first transmembrane domain would be considered dysfunctional, aGPCRs are thought to have bimodal functions [35–37]. Here, the CTF mediates the intracellular signal cascade, called ‘*cis*-signaling’ while the NTF resolves the ‘*trans*-signaling’ to other cells. Since there is no report on *ADGRD1*-deficient animal models available the individual functions and the physiological relevance of NTF vs. CTF are unknown yet.

## Conclusion

Our analysis of naturally occurring SNPs within the *ADGRD1* gene showed that this receptor displays mutation frequencies that are similar to a prototypic GPCR. While functional consequences of the nsSNPs with respect to intracellular signal transduction are highly variable, cleavage does not seem to be affected. It seems that a range between complete abolishment of up to a 3-fold increase in ADGRD1 function is compatible with human life and development at least with only one allele being affected. Gene-deficient animal models will shed light on the physiologic relevance of this evolutionarily conserved GPCR. However, the existence of clinically healthy carriers for functionally relevant *ADGRD1* variants will eventually lead to the identification of homozygous / compound heterozygous individuals exposing the phenotypic relevance also in humans.

## Methods

If not stated otherwise, all standard substances were purchased from Sigma-Aldrich (Taufkirchen, Germany), Merck (Darmstadt, Germany), and C. Roth GmbH (Karlsruhe, Germany). Cell culture material and primers were obtained from *Thermo Fisher Scientific Inc.,* (Darmstadt, Germany).

### Data base search for single nucleotide polymorphisms

Non-synonymous SNPs (nsSNPs) found in human *ADGRD1* were extracted (latest update 21-04-2015) from the NCBI database of Single Nucleotide Polymorphisms (dbSNP) [24], which combines results from 1000Genomes [13], exome sequencing projects (NHLBI-ESP [14], ClinSeq Project [15]), the human haplotype map (HAP-Map [16]) as well as smaller sequencing projects (e.g. exome, Illumina Chip). A novel nsSNP (A446V) was found by an exome sequencing study which identified alterations influencing human height [8] and was also included.

### Generation of wild type (wt) and mutant ADGRD1 constructs

The human ADGRD1 was N-terminally tagged with a haemagglutinin (HA) epitope and C-terminally tagged with a FLAG epitope based on [22]. nsSNPs were generated through site-directed mutagenesis strategy. One of the reported nsSNPs results in a frameshift and prolongs the protein by 78 amino acids (AA). To generate this frameshift mutant (C632fs), it was necessary to clone the *ADGRD1* mRNA 3′UTR region (NM_198827.3) from human leucocytes (primer: forward 5′-GTACATGTTTGCCACGCTCA-3′, reverse 5′-GATGAAGGGAGGCTCAAGGG-3′) and insert a Flag tag into the new frame in front of the new stop codon to evaluate its expression levels. One of the nsSNPs, (Q600stop), results in a premature stop codon central of the intracellular loop 1 (ICL1). To generate this mutant it was necessary to clone the FLAG-epitope upstream of the stop signal (primer: forward 5′-tacaaggatgacgacgataagTAGCGCTACCACATCCACG-3′, reverse 5′-gtcgtcatccttgtaatcGTTCCGGATGGTGCTCAC-3′, small letters indicate the FLAG sequence).

All plasmids were introduced into the eukaryotic expression vector pcDps. All generated constructs were confirmed by sequencing.

### Cell culture and transfection

COS-7 and HEK293T cells were cultured in Dulbecco’s minimum essential medium (DMEM) containing 10 % fetal bovine serum (FBS), 100 units/ml penicillin and 100 μg/ml streptomycin at 37 °C and 5 % CO_2_ in a humidified atmosphere. One day prior to transfection, COS-7 cells were split into 48-well plates (3 × 10^4^ cells/well for cell surface ELISA), or 6-well plates (3 × 10^5^ cells/well for sandwich ELISA and Western Blot). For secreted alkaline phosphatase (SeAP) and luciferase assays HEK293T cells were split into 96-well plates (3 × 10^4^ cells/well). Transfection of cells was done using Lipofectamine™ 2000 (*Thermo Fisher Scientific Inc.,*, Darmstadt, Germany) according to the manufacture’s protocol.

### Functional assays

Because ADGRD1 is a G_s_-coupled receptor function was measured with cAMP response element (CRE) secreted alkaline phosphatase (SeAP) and CRE binding protein (CREB) luciferase assays. SeAP plasmid (Takara Bio Europe SAS, Saint-Germain-en-Laye, France) and wt or mutant ADGRD1 constructs were co-transfected with each 75 ng of DNA/well. Basal and peptide agonist induced luciferase as well as SeAP levels were measured after 5 h of incubation. For SeAP experiments endogenously expressed alkaline phosphatases were heat inactivated for 2 h right before the measurement. 10 μM forskolin was used as positive control for CRE and CREB activities. As an additional readout for cAMP formation we measured luciferase activity under the control of CREB. Thus, co-transfection of 50 ng receptor plasmid and 50 ng luciferase plasmid (PathDetect trans-reporting System) was performed using HEK293T cells. Luciferase activity was detected using steadylite plus (PerkinElmer, Rodgau, Germany).

### Expression

To detect cell surface and whole cell expression in transfected COS-7 cells ELISAs were used as described previously [38].

### Data analysis

Measurements were performed at least three times in triplicates. After normalization to mock-transfection (empty vector (EV)) data were compared to wt function and expression in percentage of each trial. All amino acid positions are based on wt ADGRD1 variant NP_942122.2. Graphical and statistical analyses were done using Prism version 5.01 (GraphPad Software Inc., San Diego, CA). Statistical methods are described in the figure legends.

## Abbreviations

aGPCR, adhesion G protein-coupled receptor; CRE, cAMP response element; CREB, CRE binding protein; CTF, C-terminal fragment; dbSNP, NCBI database of Single Nucleotide Polymorphisms; DMEM, Dulbecco’s minimum essential medium; ELISA, enzyme-linked immunosorbent assay; EV, empty vector; GAIN, GPCR autoproteolysis inducing; GPS, GPCR proteolysis site; GWAS, Genome-wide association study; HA, haemagglutinin; ICL1, intracellular loop 1; LamG3, Laminin G3; nsSNP, non-synonymous SNP; NTF, N-terminal fragment; PTX, pentraxin; SD, standard deviation; SeAP, secretion of alkaline phosphatase; SNP, single nucleotide polymorphism; UTR, untranslated regions; VLDL, very low density lipoproteins; wt, wildtype

## Additional files

Additional file 1: Table S1. Here, for all inspected nsSNPs descriptive information’s can be found (e.g. Ref. SNP accession numbers, Chr. position, genotype frequencies, references) as well as raw data of the functional analysis and degree of conservation. (XLSX 38 kb) Additional file 2: Here, all suppl. tables (suppl. tab. S1-S3), figures (suppl. fig. S1 and S2) and methods (Western Blot, only used for supplementary data) are listed. (DOCX 1357 kb)
