# Supplementary material for: Functional relevance of naturally occurring mutations in adhesion G protein-coupled receptor ADGRD1 (GPR133)
Source: BMC Genomics. 2016 Aug 11;17:609. doi: 10.1186/s12864-016-2937-2 (PMC4982218; doi:10.1186/s12864-016-2937-2)
Supplement: Additional file 2: — Here, all suppl. tables (suppl. tab. S1-S3), figures (suppl. fig. S1 and S2) and methods (Western Blot, only used for supplementary data) are listed. (DOCX 1357 kb) [file 12864_2016_2937_MOESM2_ESM.docx]

**Functional Relevance of naturally occurring Mutations in adhesion G Protein-coupled Receptor ADGRD1 (GPR133)**

**Liane Fischer, Caroline Wilde, Torsten Schöneberg^#^, Ines Liebscher^#^**

From the Section of Molecular Biochemistry, Institute of Biochemistry, Medical Faculty, University of Leipzig, 04103 Leipzig, Germany

^#^Address correspondence to: I.L. (liebscher@medizin.uni-leipzig.de) or T.S. (schoberg@medizin.uni-leipzig.de), Institute of Biochemistry, Medical Faculty, University of Leipzig, Johannisallee 30, 04103 Leipzig, Germany

**Supplements**

**suppl. Methods**

*Western Blot* - Full length constructs of mutant and wild-type (WT) human GPR133 were used to detect autoproteolytic cleavage. COS-7 cells were transfected with the respective full length constructs. The culture medium was replaced with serum-reduced Opti-MEM I (Life Technologies, Darmstadt, Germany) 15 h post transfection and incubated for further 48 h. Cells were lysed and proteins were then resuspended in 2x Laemmli buffer, separated by 12.5% SDS-PAGE and transferred to nitrocellulose membranes (Amersham, Freiburg, Germany). Blots were then probed with the horseradish-peroxidase-conjugated antibody rat anti-HA (Sigma-Aldrich, Taufkirchen, Germany) following standard protocols. Membranes were cleared from staining with stripping buffer (0.2 M glycine, 0.1% SDS, 1% Tween20, pH 2.2) for 7 min, then incubated in PBS for 10 min and further incubated in TBS-T for 5 min, blocked and then probed with antibody.

suppl. Tab. S1: Available information on inspected nsSNPs in *ADGRD1* (attached as Excel file)

suppl. Tab. S2: Distribution of nsSNP function and expression compared to wt

|  | CRE | cell surface expression |
| --- | --- | --- |
| wildtype function (±1SD) | 58.9 % | 81.2 % |
| <1SD of wildtype function | 17.9 % | 17.0 % |
| <2SD of wildtype function | 11.6 % | 7.1 % |
| >1SD of wildtype function | 23.2 % | 1.8 % |
| >2SD of wildtype function | 12.5 % | 0 % |

suppl. Tab. S3: List of orthologous sequences (used for the alignment in suppl. Fig. 1)

| **Class** | **Clade** | **Organism** | **Accession** | **Accession** |
| --- | --- | --- | --- | --- |
| **Mammalia** | **Afrotheria** | *Echinops telfairi* | XP_004709805.1 |  |
|  |  | *Loxodonta africana* | XP_003421298.1 |  |
|  |  | *Trichechus manatus latirostris* | XP_004385107.1 |  |
|  |  | *Elephantulus edwardii* | XP_006901058 |  |
|  |  | *Orycteropus afer afer* | XP_007953241 |  |
|  |  | *Chrysochloris asiatica* | XP_006874143 |  |
|  | **Euarchontoglires** | *Ochotona princeps* | XP_004595482.1 |  |
|  |  | *Oryctolagus cuniculus* | XP_002722734.1 |  |
|  |  | *Cavia porcellus* | XP_003461211.1 |  |
|  |  | *Chinchilla lanigera* | XP_005403043.1 |  |
|  |  | *Octodon degus* | XP_004636012.1 |  |
|  |  | *Heterocephalus glaber* | XP_004882350.1 |  |
|  |  | *Ictidomys tridecemlineatus* | XP_005336395.1 |  |
|  |  | *Cricetulus griseus* | XP_007651841.1 |  |
|  |  | *Mesocricetus auratus* | XP_005080644.1 |  |
|  |  | *Microtus ochrogaster* | XP_005344587.1 |  |
|  |  | *Mus musculus* | XP_006504370.1 |  |
|  |  | *Rattus norvegicus* | XP_001070157.3 |  |
|  |  | *Callithrix jacchus* | XP_009003158.1 |  |
|  |  | *Saimiri boliviensis boliviensis* | XP_003937140.1 |  |
|  |  | *Gorilla gorilla gorilla* | XP_004054212.1 |  |
|  |  | *Homo sapiens* | NP_942122.2 |  |
|  |  | *Pan paniscus* | XP_003817893.1 |  |
|  |  | *Pan troglodytes* | XP_009424856.1 | XP_003314077.1 |
|  |  | *Nomascus leucogenys* | XP_003276179.1 |  |
|  |  | *Pongo abelii* | XP_002824041.2 | XP_002824042.1 |
|  |  | *Macaca fascicularis* | EHH66843.1 |  |
|  |  | *Macaca mulatta* | XP_001104579.1 |  |
|  |  | *Papio anubis* | XP_003907445.1 |  |
|  |  | *Otolemur garnettii* | XP_003802644.1 |  |
|  | **Laurasiatheria** | *Mustela putorius furo* | XP_004778256.1 |  |
|  |  | *Odobenus rosmarus divergens* | XP_004415514.1 |  |
|  |  | *Canis lupus familiaris* | XP_543354.2 |  |
|  |  | *Felis catus* | XP_003994639.1 |  |
|  |  | *Ailuropoda melanoleuca* | XP_002929658.1 |  |
|  |  | *Bos mutus* | XP_005891284.1 |  |
|  |  | *Bos taurus* | XP_005217719.1 |  |
|  |  | *Capra hircus* | XP_005691392.1 |  |
|  |  | *Pantholops hodgsonii* | XP_005973967.1 |  |
|  |  | *Ovis aries* | XP_004017353.1 |  |
|  |  | *Orcinus orca* | XP_004276681.1 |  |
|  |  | *Sus scrofa* | XP_003483456.2 | XP_003359120.2 |
|  |  | *Ceratotherium simum simum* | XP_004430136.1 |  |
|  |  | *Equus caballus* | XP_001494231.1 |  |
|  |  | *Condylura cristata* | XP_004690697.1 |  |
|  |  | *Sorex araneus* | XP_004611179.1 |  |
|  |  | *Pteropus alecto* | ELK14243.1 |  |
|  | **Xenarthra** | *Dasypus novemcinctus* | XP_004455622.1 |  |
| **Aves** |  | *Anas platyrhynchos* | XP_005018133.1 |  |
|  |  | *Gallus gallus* | XP_415094.4 |  |
|  |  | *Meleagris gallopavo* | XP_003210929.1 |  |
|  |  | *Columba livia* | XP_005498339.1 |  |
|  |  | *Falco cherrug* | XP_005445260.1 |  |
|  |  | *Falco peregrinus* | XP_005233359.1 |  |
|  |  | *Melopsittacus undulatus* | XP_005147783.1 |  |
|  |  | *Ficedula albicollis* | XP_005055238.1 |  |
|  |  | *Geospiza fortis* | XP_005424047.1 |  |
|  |  | *Taeniopygia guttata* | XP_002187734.2 |  |
|  |  | *Pseudopodoces humilis* | XP_005524268.1 |  |
| **Fish** |  | *Danio rerio* | XP_003199178.2 |  |
|  |  | *Haplochromis burtoni* | XP_005927012.1 |  |
|  |  | *Maylandia zebra* | XP_004544189.1 |  |
|  |  | *Pundamilia nyererei* | XP_005729618.1 |  |
|  |  | *Oreochromis niloticus* | XP_005473142.1 |  |
|  |  | *Takifugu rubripes* | XP_003974557.1 |  |
|  |  | *Oryzias latipes* | XP_004072772.1 |  |
|  |  | *Xiphophorus maculatus* | XP_005806977.1 |  |
|  |  | *Latimeria chalumnae* | XP_005987240.1 |  |
| **Reptilia** |  | *Alligator sinensis* | XP_006017140.1 |  |
|  |  | *Chrysemys picta bellii* | XP_005299599.1 |  |
|  |  | *Pelodiscus sinensis* | XP_006120595.1 |  |
|  |  | *Chelonia mydas* | XP_007055034 |  |
|  |  | *Alligator mississippiensis* | XP_006268019 |  |
|  |  | *Python bivittatus* | XP_007426722 |  |
| **Amphibia** |  | *Xenopus tropicalis* | XP_002931944.2 |  |

**suppl. Fig. S1: Degree of conservation correlates with signaling activity**. Alignment over consensus sequences of different orthologous arranged in classes (Aves, Fish, Reptilia and Amphibia) or clades in case of mammalia (Euarchontoglires, Laurasiatheria, Afrotheria, and Xenarthra) of *ADGRD1*. To create consensus sequences a threshold of 50 % was assumed. The numbers of aligned sequences are 11 for Aves, 9 for Fish, 6 for Reptilia, *Xenopus tropicalis* for Amphibia, 24 for Euarchontoglires, 17 for Laurasiatheria, 6 for Afrotheria, and *Dasypus novemcinctus* for Xenarthra. For accession numbers please see suppl. Tab. S3. Functional relevant nsSNPs (± 2SD) (M1T, E78K, Y85C, F110L, G141D, W174S, E178K, G195R, F383S, V393M, G404A, P411S, E413K, A448D, D453N, S667L, A761E, V764M, N795K, A816T, T827M) are tagged with plus for constitutive active variants or a minus for basally less active nsSNPs. Compartments like signal peptide (SP), pentraxin/laminin G3 domain (PTX/LamG3), GPCR autoproteolysis inducing domain (GAIN) with subdomain A and B including the GPCR proteolysis site (GPS) and 7TM region (I-VII) are shown. Black-shaded fields show identical characters (threshold 100 %) whereas gray-shaded fields show similar characters produced with the BLOSUM62 matrix. Alignments were generated using ClustalW in BioEdit 7.1.11 [1].

**suppl. Fig. S1**


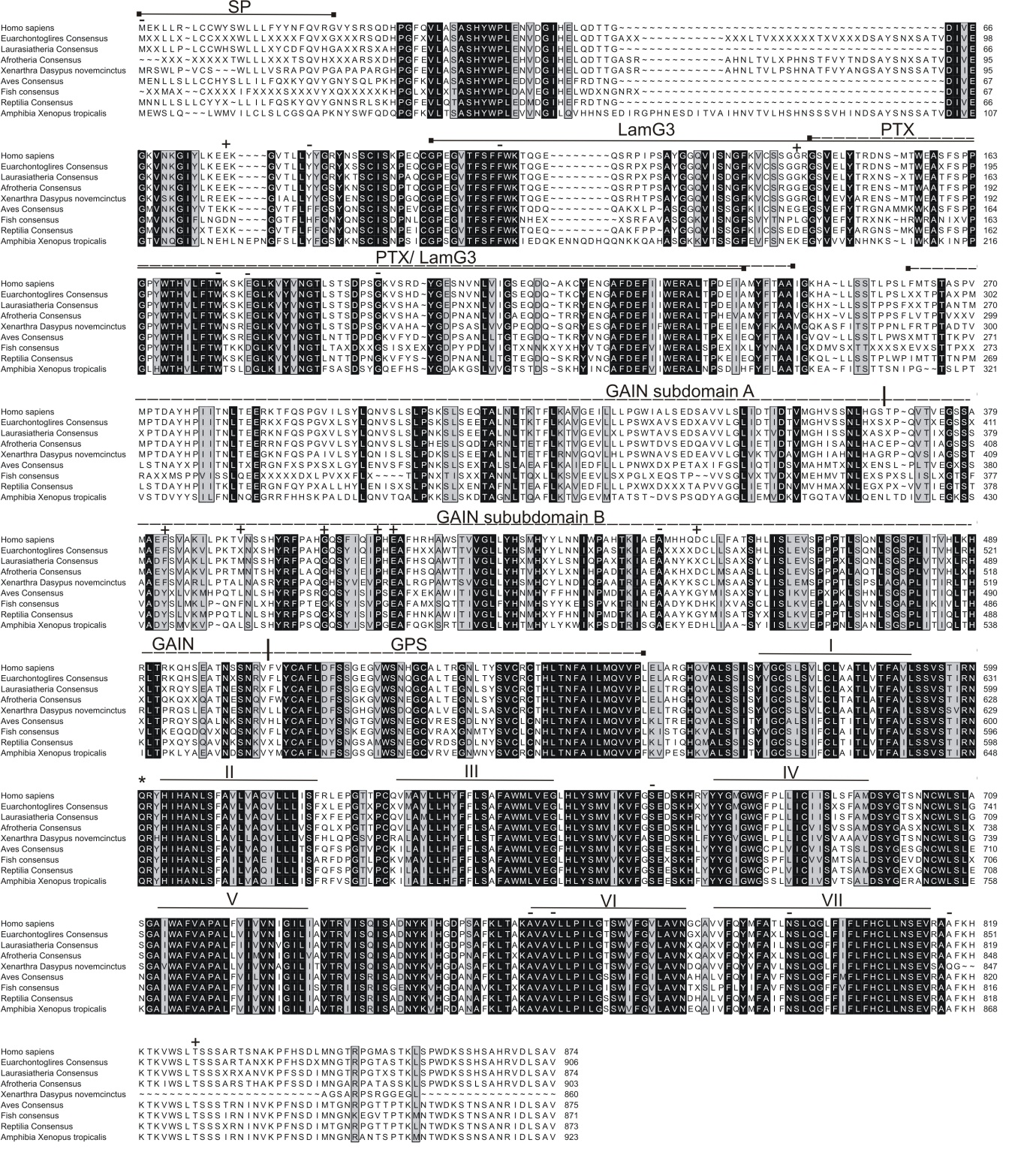


**suppl. Fig. S2: Cleavage analysis of selected SNPs.** Western blot results of cell lysates detecting the N-terminal HA-tag of full-length GPR133. All analyzed SNPs showed bands between 80-100 kDa, due to different glycosylation rates of the NTF. These bands have been shown to be absent in the cleavage-deficient GPR133 mutant H540R [2].


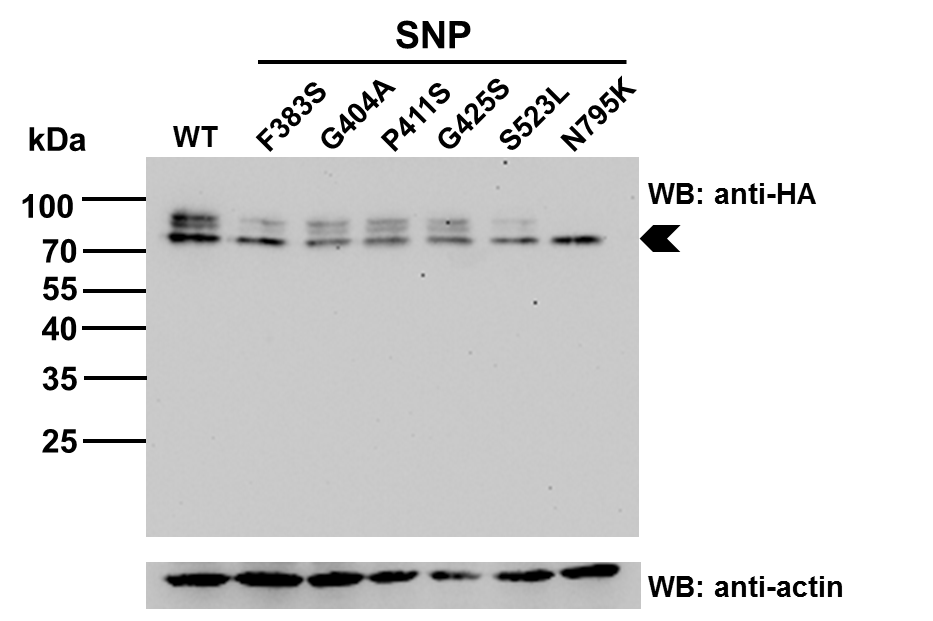


References

1. Hall TA: **BioEdit: a user-friendly biological sequence alignment editor and analysis program for Windows 95/98/NT.** *Nucl. Acids. Symp. Ser.* 1999, **41**:95-98.

2. Liebscher I, Schön J, Petersen SC, Fischer L, Auerbach N, Demberg LM, Mogha A, Cöster M, Simon K, Rothemund S, Monk KR, Schöneberg T: **A Tethered Agonist within the Ectodomain Activates the Adhesion G Protein-Coupled Receptors GPR126 and GPR133**. *Cell reports* 2014, **9**:2018-2026.
